# Dietary phytochemicals and their role in cancer chemoprevention

**DOI:** 10.20517/2394-4722.2021.125

**Published:** 2021-06-08

**Authors:** Shashank Kumar, Sanjay Gupta

**Affiliations:** 1 Molecular Signaling and Drug Discovery Laboratory, Department of Biochemistry, School of Basic and Applied Sciences, Central University of Punjab, Bathinda 151401, India.; 2 Department of Urology, Case Western Reserve University, Cleveland, OH 44106, USA.; 3 Department of Urology, The Urology Institute, University Hospitals Cleveland Medical Center, Cleveland, OH 44106, USA.; 4 Department of Nutrition, Case Western Reserve University, Cleveland, OH 44106, USA.; 5 Divison of General Medical Sciences, Case Comprehensive Cancer Center, Cleveland, OH 44106, USA.; 6 Department of Urology, Louis Stokes Cleveland Veterans Affairs Medical Center, Cleveland, OH 44106, USA.

Epidemiological studies suggest a close association between diet and cancer initiation, which provides evidence that the dietary components may be effectively developed as chemopreventive agents^[1]^. These pieces of evidence are further supported by several case-control and cohort studies, which overwhelmingly support a converse association between the intake of phytochemicals and cancer risk^[2,3]^. A number of clinical studies have been conducted demonstrating that dietary phytochemicals have the ability to inhibit tumorigenesis^[4]^. Components present in fruit and vegetables termed “bioactive” phytochemicals belong to several classes of micronutrients, including flavonoids, polyphenols, and dietary fiber. These components have the ability to reduce the cancer risk alone or by interactions between them.

Cancer chemoprevention is a means to control, slow, or reverse of cancer occurrence by administrating one or more synthetic or naturally occurring compounds^[5]^. Chemoprevention also implies the prevention of precancerous lesions, such as pre-invasive neoplasia, dysplasia, or intraepithelial neoplasia, depending on the organ system^[5]^. Therefore, phytochemicals serve as chemopreventive agents that could be beneficial for the prevention of all aspects of carcinogenesis.

The special issue on “Phytochemicals and Cancer Chemoprevention” contains original research and review articles is aimed to stimulate the continuous efforts to understand the identification, development and use of phytochemicals for cancer prevention and treatment. It has been estimated that more than two-thirds of human cancers are preventable through proper diet and lifestyle changes. In 1981, Doll and Peto^[6]^ reported that 30%−40% of human cancer is preventable, and its mortality is attributed to diet. Their observations were based on statistical and epidemiological data with major concerns on dietary factors that increase cancer risk^[6]^. Although the exact percentage is uncertain, compelling evidence from epidemiological, clinical, and laboratory studies suggest a link between cancer risk and nutritional factors.

Several phytochemicals have been identified as bioactive agents which possess cancer preventive and therapeutic properties. Dave *et al*.^[7]^ reviewed some well-established natural phytochemicals as cancer chemopreventive agents, including resveratrol (grapes), epigallocatechin-3-gallate (green tea), sulforaphane (cruciferous vegetables), anthocyanins (grapes and berries), curcumin (turmeric), silibinin (milk thistle), and lycopene (tomatoes). As appropriately demonstrated by the genomic analysis and other methods, the mechanistic underpinning remains variable and complex. The authors further suggest that these responses may be mediated through indirect mechanisms, including interaction with the microbiome. In addition, ancillary applications of the chemopreventive-agents remains worth considering, such as the management of chemotherapy induced sequelae. Recognizing the loss of millions of cancer patients every year, it is obvious that negating malignant metastatic conditions remains of paramount importance. In meeting these goals, cancer chemoprevention by phytochemicals offers great promise.

The importance of phytochemicals lies in their superior properties of reducing side-effects, efficacy in chronic conditions, cost-effectiveness, and widespread usage of natural compounds. The manuscript by Kushwaha *et al*.^[8]^ has proposed using phytochemicals in cancer chemotherapy having a promising future. Through *in silico* approach, the authors have shown that the phytochemicals present in *Bulbine frutescens* (*Asphodelaceae*) are involved in the mitigation of anticancer drug-mediated resistance. Through an approach utilizing molecular docking and molecular dynamics simulation, the authors have screened 25 phytochemicals against the ABC transporter protein. The involvement of drug efflux transporters such as P-glycoprotein, also known as ABC (ATP-binding cassette) transporters, facilitates the transport of drugs and their metabolites across the cellular membrane of cancer cells. ABC transporter-mediated drug efflux causes disease relapse in cancer patients and decreases the therapeutic output; therefore, it remains a putative drug target. The authors conclude that 4’-Demethylknipholone 2’-β-D-glucopyranoside, a phytochemical present in *B. frutescens* possesses remarkable anti-drug resistance properties and inhibitory potential against ABC transporters.

Another review article published by Yeger and Mokhtari^[9]^ discussed the isothiocyanates family of naturally occurring small molecules generated from the glucosinolate precursors of cruciferous vegetables. The cancer chemopreventive roles of some key isothiocyanates such as sulforaphane, phenethylisothiocyanate, and benzyl isothiocyanate have been studied in preclinical and clinical settings. In the review article, the authors have presented a broader perspective on the isothiocynates, including their research on sulforaphane-mediated cancer prevention and treatment. The authors further discussed the hormetic effects which might affect the efficacy of these agents, and their role as anti-inflammatory molecules with insights into potential therapeutic applications.

The review article by Grynkiewicz^[10]^ discussed the research on isoflavones in human health. Flavonoids are a large family of polyphenols that include over 6000 members widely distributed in the plant kingdom. Isoflavones, a subfamily of flavonoids, is the main focus of the review. These compounds are major constituents present in soybeans, soy foods, and legumes possessing antioxidant, antimicrobial, and anti-inflammatory properties. Natural isoflavones belong to the phenylpropanoid category and share considerable chemical and biochemical characteristics as they belong to similar biogenetic pathways. In the review, the author discussed the pharmacology and estrogenic nature of isoflavones. An attempt was also made to mention the hurdles faced in clinical research on isoflavones due to their low solubility and bioavailability. The authors further highlighted new advances in isoflavone research by chemical derivatization and developed a suitable bioavailability-enhancing formulation for further research.

The special issue concludes with the article by Wenner *et al*.^[11]^, discussing the molecular mechanisms of chemoprevention elicited by the phytochemicals against various diseases, including cancer. The authors discussed the cellular process of autophagy and an evolutionarily conserved self-digestion process that employs lysosomal-mediated enzymatic degradation. The authors discussed the autophagy induction potential of phytochemicals such as phenethyl isothiocyanate, capsaicin, withaferin A and genistein. Molecular pathway context dependent cytoprotective/cytotoxic role of autophagy and its role in cancer chemoprevention were also discussed. Understanding the pharmacology of these phytochemicals used alone or in combination with other conventional therapies, could lead to the development of new approaches in health promotion and disease prevention.
